# What do review papers conclude about food and dietary patterns?

**DOI:** 10.3402/fnr.v57i0.20523

**Published:** 2013-03-04

**Authors:** Elisabet Wirfält, Isabel Drake, Peter Wallström

**Affiliations:** Research group in Nutritional Epidemiology, Department of Clinical Sciences in Malmö, Lund University, Malmö, Sweden

**Keywords:** systematic review, whole diet, food patterns, indices, methodology, chronic disease

## Abstract

Nutrients and other bioactive constituents of foods may interact with each other and the surrounding food matrix in complex ways. Therefore, associations between single nutrients and chronic disease may be difficult to identify and interpret, but when dietary patterns (DPs) are examined the combination of many food factors will be considered. An explorative literature search of published review articles was conducted to obtain a fuller understanding of current DPs in epidemiological research, to discuss pros and cons of DPs in nutrition research, and to identify results of studies linking DPs to chronic disease risk in adults. Randomized feeding trials providing the experimental diets to study participants have repeatedly demonstrated that diets based on current dietary recommendations are associated with important health benefits. Systematic reviews of feeding trials and prospective population studies of DPs and chronic disease risk reach similar conclusions regardless of the methodology used to construct DPs. However, to date only a few review articles of DP studies have followed a systematic process using independent reviewers with strict inclusion, exclusion, and study quality criteria. Diets with plenty of plants foods, fish, and seafood that preferably include vegetable oils and low-fat dairy products are associated with a lower risk of most chronic diseases. In contrast, Western-type DPs with food products low in essential nutrients and high in energy, like sugar-sweetened beverages, sweets, refined cereals and solid fats (e.g. butter), and high in red and processed meats, are associated with adverse health effects. An emphasis on high-quality original research, and systematic reviews following a structured process to objectively select and judge studies, is needed in order to enforce a strong future knowledge base regarding DPs and chronic disease.

Experimental, clinical, and epidemiological nutrition research has traditionally strived to identify the specific mechanisms and health effects of single nutrients. However, because each food item contains energy, essential nutrients, and a multitude of bioactive substances that interact with each other and the surrounding food matrix in complex ways, the search for associations between single food factors and chronic disease may be difficult and confusing (1–4). It is argued that nutrition studies choosing a traditional nutrient specific approach may underestimate the total health impact of natural foods, and could lead to inaccurate interpretations of study outcomes resulting in the formulation of erroneous dietary advice (2, 4, 5).

The very high dosages and different chemical forms of nutrients contained in supplements, compared to the nutrients in natural foods, likely contribute to the disappointing results of randomized clinical trials, initiated to confirm the dietary importance of nutrients like beta-carotene or vitamin E (4). Studies also report that supplement use may have adverse health effects (6). In contrast, carefully conducted intervention studies have demonstrated the efficiency and advantage of whole diet trials when searching for preventive strategies to chronic disease (4, 5, 7, 8).

The increasing awareness of difficulties when searching for the impact of single nutrients in nutrition epidemiology have stimulated researchers to explore the health effect of food item combinations that is food or dietary patterns (DPs) (9–12), and to argue for a more holistic approach to nutrition research for biological as well as statistical reasons (2, 4, 10, 11, 13–16). However, with accumulating experiences, researchers have raised problematic methodological issues when constructing DPs (5, 17–20). Others debate whether more refined approaches are needed considering the complexity of diet and differences in disease etiologies (2, 21, 22). In order to obtain a fuller understanding of the current view of DPs in epidemiological research using either data-driven methods or dietary indexes, the purpose of this report was to explore review articles regarding DP studies. The specific aims were to discuss the pros and cons of DP studies, including considerations of dietary complexities and methodological issues, and to identify and discuss the results of systematic review articles of studies among adults linking DPs to chronic disease risk.

## Methods

The present literature review is a part of the project reviewing and updating the scientific basis in preparation for the fifth version of the Nordic Nutrition Recommendations (NNR). The foci of the NNR project are those areas where new scientific knowledge has emerged since the fourth edition, with special relevance for the Nordic setting. A number of systematic literature reviews (SR) will form the basis for establishment of dietary reference values in the fifth edition of NNR. However, the literature review presented in the current paper is not a SR, but the result of an explorative literature search based on the research questions and the search string displayed in Table 1.

**Table 1:** Literature search conducted to identify review articles on food and dietary patterns, published 2000–2011. Summary of search: task/research question, MESH identifiers and search string

Task:
1. To identify Review articles
2. To include articles that use data-driven food or dietary patterns (constructed using factor analysis, cluster analysis or reduced rank regression)
3. To include articles that use index based dietary patterns (e.g. healthy diet or Mediterranean diet indices)

MESH identifiers:
food pattern*[Title/Abstract] **OR**
dietary pattern*[Title/Abstract] **OR**
eating pattern*[Title/Abstract] **OR**
‘Diet, Mediterranean’ [Mesh] **OR**
‘Diet/adverse effects’ [Mesh] **OR**
‘Diet/standards’ [Mesh] **OR**
Food habits[Mesh] **OR**
‘Food habits/classification’ [Mesh] **OR**
‘Food habits/ethnology’ [Mesh] **OR**
‘Food habits/physiology’ [Mesh] **OR**
‘Food preferences’ [Mesh]
**AND**
‘Index based’ [Title/Abstract] **OR**
‘Data driven’ [Title/Abstract] **OR**
‘Cluster analysis’ [Mesh] **OR**
‘Factor analysis, statistical’ [Mesh] **OR**
‘Reduced rank regression’ [Title/Abstract] **OR**
‘Nutrition surveys’ [Mesh] **OR**
‘Research design’ [Mesh] **OR**
‘Epidemiologic research design’ [Mesh] **OR**
‘Epidemiologic methods’ [Mesh] **OR**
‘Health status indicators/methods’ [Mesh] **OR**
‘Health status indicators/standards’ [Mesh] **OR**
‘Health status indicators/trends’ [Mesh] **OR**
‘Diet/statistics and numerical data’ [Mesh])
**AND**
‘Review’.[Publication Type] **AND**
‘English’ **AND**
‘Humans’ [Mesh] **AND**
‘2000/01/01’ [Publication Date]: ‘2011/01/31’ [Publication Date]
Search string, all identifiers listed as above:
(food pattern*[Title/Abstract] **OR** dietary pattern*[Title/Abstract] **OR** eating pattern*[Title/Abstract] **OR** ‘Diet, Mediterranean’ [Mesh] **OR** ‘Diet/adverse effects’ [Mesh] **OR** ‘Diet/standards’ [Mesh] **OR** Food Habits [Mesh] **OR** ‘Food Habits/classification’ [Mesh] **OR** ‘Food Habits/ethnology’ [Mesh] **OR** ‘Food Habits/physiology’ [Mesh] **OR** ‘Food Preferences’ [Mesh]) **AND** (‘index based’ [Title/Abstract] **OR** ‘data driven’ [Title/Abstract] **OR** ‘Cluster Analysis’ [Mesh] **OR** ‘Fact**OR** Analysis, Statistical’ [Mesh] **OR** ‘reduced rank regression’ [Title/Abstract] **OR** ‘Nutrition Surveys’ [Mesh] **OR** ‘Research Design’ [Mesh] **OR** ‘Epidemiologic Research Design’ [Mesh] **OR** ‘Epidemiologic Methods’ [Mesh] **OR** ‘Health Status Indicators/methods’ [Mesh] **OR** ‘Health Status Indicators/standards’ [Mesh] **OR** ‘Health Status Indicators/trends’ [Mesh] **OR** ‘Diet/statistics **AND** numerical data’ [Mesh]) **AND** (‘Review’ [Publication Type]) **AND** (‘English’) **AND** (‘Humans’ [Mesh]) **AND** (‘2000/01/01’ [Publication Date] : ‘2011/01/31’ [Publication Date])

**Fig. 1:**
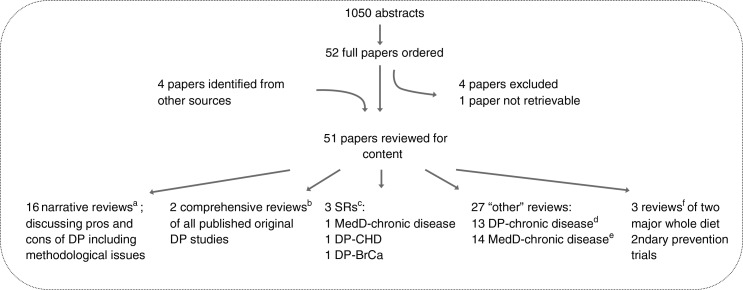
Flow-chart of the literature search regarding review papers of original research articles of food or dietary patterns published from 2000 to 2010. DPs=food and dietary patterns (using both *a priori* and *a posteriori* methods); SR=systematic literature review; MedD=Mediterranean-like dietary pattern; CHD=coronary heart disease; BrCa=breast cancer.

^a^(2, 5, 7, 10, 14–16, 18–20, 22, 29–33), ^b^(11, 34), ^c^(60–62), ^d^(3, 28, 35–45), ^e^(46–59), ^f^(7, 8, 64).

In total, this search identified 1,050 potential abstracts, out of which 52 specifically mentioned DPs (see Fig. 1). After closer scrutiny of the full articles of these abstracts, four papers were excluded that neither reviewed DP studies nor discussed issues related to DP studies (23–26). Also, one eligible paper was not accessible and had to be excluded (27). An additional four review papers of DP studies were identified from other sources (5, 16, 20, 28). In total, 51 articles were examined for content and are discussed in this report. These articles are identified in Fig. 1 in the following order: Narrative reviews; Comprehensive reviews of all published DP studies; SRs of DPs, or Mediterranean-like dietary patterns (MedD), and chronic disease risk; ‘Other’ reviews of DPs or MedD, and chronic disease risk; and reviews of major whole diet secondary prevention trials.

The majority of review papers identified in the literature search did not focus on specific endpoints, but were general or narrative reviews discussing the rationale for the DP approach in nutrition research, or describing the process of constructing DPs, or discussing methodological issues regarding *a priori* or *a posteriori* DP approaches. These articles are cited in several sections of this paper, but are mostly found under the heading ‘DPs in observational studies’ (2, 5, 10, 13–16, 18–20, 22, 29–33).

Two articles were comprehensive reviews of published DP studies (i.e. in 2004 or earlier) in observational epidemiology without focusing on any specific endpoint (11, 34).

Thirteen articles reviewed original studies of associations between DPs and chronic disease risk (3, 28, 35–45). Another 14 articles specifically targeted the health effects of the MedD (46–51) (52–59). Although several of these 27 papers identified themselves as ‘systematic review or search’, only three papers were SRs in a strict sense, utilizing independent reviewers; specified the criteria for study quality assessment; presented pooled risk estimates (meta-analysis); and tested heterogeneity and publication bias across studies (60–62). Thus, these three papers are SRs as defined by the guidelines found in the manual of the NNR revision project (63). These articles are described in Table 2 in more detail.

**Table 2:** Three systematic review^a^ articles (60–62), identified through literature search of dietary patterns and chronic disease of articles published 2000–2011

Author (year)	Study focus	Type of studies reviewed	Years covered	Criteria	Conclusions	Comments regarding methodology
Mente (2009)	Dietary factors and coronary heart disease	5705 potential articles; Reviewed: 361 cohort studies and 51 RCT	1950–2007	Several dietary exposures; CHD and fatal or non-fatal MI; Bradford Hill criteria: strength, consistency, temporality and coherence	Strong protection: Vegetables, nuts, MUFA, MedD, prudent and High-quality DPs; Strong harmful effects: trans-fat, high GI/GL and Western DPs; RCT only examined Med-diet	Two independent reviewers; complicated and extensive evaluation; pooled analyses; heterogeneity; stratified analyses
Brennan (2010)	Dietary patterns and breast cancer	73 articles identified; Reviewed: 16 articles of 18 case-control and cohort studies	2001–2009	Diet and breast cancer risk; Three patterns: Western/unhealthy, prudent/healthy, and drinker; FFQ (2 DH)	Prudent/healthy DPs protection; drinker DPs increased breast cancer risk; Western pattern only significant in case-control studies, suspected bias	Three independent reviewers; pooled analysis; publication bias, heterogeneity, sensitivity analysis
Sofi (2010)	Mediterranean diet and health	18 cohort studies identified and reviewed	1966–June 2010	Prospective studies; MedD score; adverse outcomes (mortality, mortality/incidence of CVD and cancer, incidence of neurodegenerative diseases)	Confirm results from a previous meta-analysis: MedD protection for overall mortality and incidence of several chronic diseases	Two independent reviewers; study quality assessment; pooled analysis; heterogeneity; publication bias; Meta-regression analysis

a Systematic review using independent reviewers, and objective criteria to select and judge studies; a process similar to that adopted for the systematic literature reviews conducted in preparation for the fifth edition of the Nordic Nutrition Recommendations.

In addition, although most articles discussed studies from observational epidemiology, a few articles identified by the literature search presented and discussed the Dietary Approaches to stop Hypertension (DASH) trials and the Lyon Heart Study, two well-designed clinical trials of whole diets (7, 8, 64). These are discussed under the section ‘Whole diet trials’.

## DPs in observational studies

Because individuals consume combinations of foods, nutrient intakes are inter-correlated, which causes problems in statistical analysis and in the interpretation of nutritional epidemiology studies (10, 11). When strongly correlated nutrient variables are entered simultaneously in the same statistical model, the independent variation of a single nutrient is markedly reduced; as a result associations with disease endpoints in epidemiological studies tend to be attenuated (10). On the other hand, when examining each nutrient variable separately (i.e. when conducting many statistical tests), the likelihood of observing statistically significant associations by chance is enhanced. Also, since single nutrient analysis does not take complex synergistic interactions into account, the dietary health effect of a single nutrient may be too small to detect. When DPs are examined, however, many dietary components in the same pattern could result in sufficiently large cumulative effects that would enhance detection. Also, when examining single nutrients there is always some risk of confounding by other nutrients of food factors found in the same dietary pattern. Thus by constructing and analyzing food patterns the co-linearity of foods is turned into an analytical advantage, while simultaneously enhancing the analytical ability to observe the total health impact of diet (10). However, researchers also recognize that chronic diseases have many facets and complex etiologies and that the scrutiny of the pathophysiological processes is difficult (2, 43). Since chronic diseases are not single nutrient deficiencies, it may be virtually impossible to pinpoint single nutrients to these processes, especially in epidemiological studies (2, 13–16).

### *A priori* methodologies

Dietary indices or scores were the first methods used in epidemiology to assess how the combination of foods or nutrients based on predefined criteria was related to health outcomes. Typically, a dietary index is based on current nutrient intake recommendations and/or dietary guidelines. A set of components is identified so that a broad aspect of the dietary recommendations is covered. Depending on the consumption level, individuals are scored on each component, and a summary score is computed for each individual so that high scores reflect dietary intakes in line with the recommended diet. These hypothesis-oriented approaches are known as *a priori* defined DPs. See also Table 3.

**Table 3:** Characteristics of *a priori* and *a posteriori* approaches to dietary pattern construction^a^

	Research question	Characteristics	Researcher decisions required
*A priori*			
Index	Assesses adherence to dietary guidelines	Ranks individuals with low scores (low quality diets) versus those with high scores (high-quality diets)	Dietary items to include Energy adjustment of dietary items Number of items to include
		Individuals with medium scores have a mix of many different exposures	Select cut-off values, or determine scales
		A gradient is formed	Select scoring approach Weighting of items to predict specific disease
*A posteriori*			
Factor	Identifies dietary practices	Factors are scales, based on the correlations among many foods	Collapsing original dietary data
		Individuals have low, medium or high values of factor scores	Avoid collapsing data to increase the statistical power
		A gradient is formed	Treatment of input variables (standardization or energy adjustment)
Cluster	Identifies dietary practices	Large clusters represent behaviors shared by many; small clusters represent very specific behaviors shared by a few individuals (outliers)	Identify patterns to report
		Food choices common to most individuals contribute little to cluster formation	Identify patterns to analyze further
		Clusters are categories where the variation of individuals is not considered after classification	Interpret and label patterns
		No gradient is formed; less powerful in statistical analysis	

a Adapted from Reedy et al. (12).

Published studies using dietary indices were first reviewed by Kant in 1996 (65) and later in a more extensive review in 2004 (34). The first paper examined an array of diet quality indices (from 56 published papers) and concluded that in epidemiological studies these indices were more strongly related to disease risk than individual nutrients or foods (65). This suggested that indices would deal with the multi-co-linearity of dietary variables, and also with the complexity of foods as environmental exposures. It was suggested that the indices needed to be validated against biochemical, anthropometrical, and clinical variables. As indicated below, more recent studies include results from such validation projects (32, 20).

The extensive review published by Kant in 2004 included the majority of published articles of DP studies, including articles using dietary variety scores, dietary recommendation indices, or Mediterranean diet indices (32 articles), and data-driven DP methodologies (47 articles) (see also below). This review concluded that the associations between indices and nutrient or biological outcome variables were in the expected directions in cross-sectional studies (34). High (healthful) index scores showed in prospective and case-control studies protective associations with all-cause mortality and cardio vascular disease (CVD) risk. However, inconsistences were observed across studies. Comparisons were difficult, because of differences in study designs and in the control of confounding from lifestyle behaviors. The conclusions were similar in a follow-up review of more recently published studies (20). Here it was concluded that inverse associations were observed between high index scores and cancer incidence and mortality in American, but not in European cohorts. Comparisons across studies were still considered difficult due to differences in study design and dietary assessment.

Two review articles have specifically focused on and discussed methodological issues of *a priori* methodologies: how the index is constructed and what it reflects (18) and how the study design may influence the outcome (33).

Waijers et al. (18) critically reviewed studies using 20 distinct dietary quality indices. The four original diet quality scores are the health eating index (HEI), the dietary quality index (DQI), healthy diet index (HDI), and the Mediterranean diet score (MDS). Current dietary guidelines are the basis for the HEI, DQI, and HDI, while the MDS has many variations depending on the populations examined. The review focused on the extreme complexity of dietary scores and many issues still unresolved and the actual composition of indices and the rationale behind them. Also, the review points to the many choices required when creating an index, which is of major concern for its usefulness and validity. The choices include: the variables to include (i.e. many items will provide overlapping information), the cut-off values to select, and how to score the included items (Table 3). For instance, many indices have used single cut-off values (e.g. medians) and created dichotomies (i.e. 1=adherence; 0=non-adherence). Such small scales may result in low discriminating power, because many individuals accumulate in one category of the index. Instead of using simple cut-offs (i.e. dichotomies), scoring ranges should be proportional to intakes (i.e. reflect the distribution) and thus ensure wider diversity and allow u-shaped health/disease associations to emerge. Although different index components likely have different health impact, it is very difficult to make statements on the relative health contribution of individual nutrients, or to introduce weighting of certain items. Any attempt of weighting index components will ignore the interaction between components, which is the specific reason why indices are used instead of single items. A follow-up review focused on recent developments and indices for specific groups and dietary diversity (29). Here, the authors also emphasize that the specific purpose of the index needs to be clear (i.e. dietary quality measure or disease predictor), and it should be tailored to the specific population. Aspects that still need further research include: ways to optimize how components are selected, combined and scored into one index/measure, and how to handle different energy intakes.

Because dietary habits are culturally determined, an index needs to reflect such characteristics. However, population differences make it difficult to compare index scores across populations, especially if the median intake is used as a cut-off (e.g. in the MDS). For instance, it is debatable whether MDS should/could be calculated for Northern Europeans. If so, individuals with high index scores in Northern Europe will have considerably lower vegetable intakes compared to individuals with high index scores in Mediterranean countries (18). A review of different indices to evaluate adherence to the MedD, from several countries (i.e. Greece, Denmark, China, Spain, other European countries, and Israel) concluded that the majority did not measure the adherence to a universal Mediterranean diet, but a pattern based on the distribution of selected food groups in the examined population (48).

The paper by Wirt (33) systematically reviewed studies using dietary quality indices published after the review by Kant (34). In total, 28 relevant articles that described 25 indices were identified. This article summarizes study design issues that need attention when the adherence to dietary guidelines is examined, and the subsequent associations with chronic diseases are evaluated. Gender differences are often observed; this could be a real disparity, or due to bias in the dietary assessment. Many indices are designed to measure adherence to dietary guidelines, not specifically to predict disease outcome. However, since dietary guidelines generally address the major health problems in industrialized countries, it is not surprising that dietary quality indices more often predict CVD risk than cancer risk. Many indices likely lack sensitivity for diet-cancer risk associations. Also, since longer follow-up periods would be needed for cancer outcomes, this observation may also depend on study duration. The authors point out that with a small scoring scale the index may not be sensitive enough, or have enough power to detect disease associations. Other study limitations include small cohort size, cross-sectional design with no follow-up, and mortality as the sole outcome. The authors conclude that studies often lack sufficient description of other design features, and clearly need to indicate if the index is intended as a global indicator of adherence to dietary guidelines, or a predictor of disease risk. Given the purpose an appropriate scoring system needs to be identified, and appropriate confounders measured. In contrast to the previous review (by Waijers et al.), weightings of index items are suggested to improve disease prediction. Weighting has also been suggested by others (22), but without concretizing how it should be implemented. Validation of the index against objective dietary biomarkers as well as disease indicators is recommended (33).

### *A posteriori* methodologies

Food or dietary patterns that are defined by using statistical analysis once the dietary data has been collected, are called *a posteriori* (or data-driven) DPs. These methods were originally developed as tools for data reduction in statistical analysis when handling large data sets with many variables, and have during the last 30 years been applied to dietary datasets in nutrition epidemiology. The DPs derived with *a posteriori* methods reflect the diets selected and consumed by individuals. Some of these DPs might have characteristics in line with current dietary recommendations, but could simultaneously incorporate dietary components of less health value. Depending on the dietary habits at hand, the *a posteriori* derived DPs will likely differ between studies and populations. See also Table 3.

Principal component analysis (PCA), or the very similar factor analysis (FA), is a widely used technique in social sciences with two distinct purposes: data reduction and theory building. The technique characterizes the covariance among many variables in terms of a few unobservable factors. In nutrition epidemiology, it aims to construct linear combinations of food intakes, which explain a high proportion of the variation in food intakes. Correlated variables are aggregated into factors that are recognized distinct from those variables, with which they are not correlated (10, 11, 16). In FA, the first factor incorporates the maximum variability; these foods are then removed and the second factor will maximally explain the remaining variance. After orthogonal rotation, factors are not correlated and may be examined in the same regression model. However, because all individuals have scores on every factor (i.e. low, medium, or high scores), the overall DP of an individual is represented by their scores on all factors. These continuous variables only reflect one aspect of an individual's diet and do not provide an intuitive picture of what exactly is consumed (i.e. the whole diet). Thus, additional analysis is needed (e.g. rank ordering of individuals on factor scores) if the purpose is to describe the DP in the population.

Reduced rank regression (RRR) is similar to PCA but works with two sets of variables (21, 39). It aims at describing linear combinations of variables belonging to one set of variables (predictor variables) by maximizing the explained variation in variables of the other set of variables (response variables). If the response variables are strong bio- or risk-markers of the disease under study, the RRR procedure should identify DPs that are related to the disease (5, 39). Since the RRR-derived DPs are not behavioral patterns (but may be proxy markers for the risk-markers), their utility beyond generating hypotheses linking food use to chronic disease is not clear. Also, comparisons across studies could be difficult, because the confounders (of both diet and disease) may vary considerably between populations and may be difficult to control (5).

If individuals need to be assigned to a certain DP, cluster analysis (CA) is the choice (11). Similar to FA, CA is a statistical data reduction tool developed in social sciences, but has been used in biological sciences and lately in molecular biology. CA aggregates individuals into maximally separated clusters based on the Euclidean distances. The Ward's method is designed to minimize the variance within clusters; the K-means method is non-hierarchical and iterative and designed to create the most distance between clusters (11). The latter has often been used in nutritional epidemiological studies, because it can handle a large number of input variables efficiently. The emerging clusters are mutually exclusive. Since one individual belongs to only one cluster these DPs are intuitively easy to handle, but as categorical variables clusters can only be examined using a reference group. The result is limited statistical power, a limitation when comparing subgroups with health outcomes. As a consequence, FA has been more common in studies examining DPs and disease outcomes (11). However, when describing a population regarding the dietary subgroups, CA is the choice and is very suitable when, for instance, designing a dietary intervention project (5).

In a comprehensive review, Newby and Tucker examined a majority of published studies (FA 58 articles; CA 35 articles) using data-driven DP methodologies (11). The aim was to report on major findings, to discuss methodological issues, and to compare the utility of FA and CA approaches. Although inconsistencies were observed, the review indicated that DPs are associated with many socioeconomic and lifestyle factors and are part of the overall lifestyle. ‘Healthy’ or prudent patterns, although with slightly different food and nutrient composition across populations, were generally associated with less disease, smaller body mass index (BMI), less mortality, and fewer cancers regardless of methodology. The need for gender stratification in DP analysis was emphasized. Follow-up reviews of more recently published DP studies in North America and Europe generally report that healthy or prudent DPs are associated with a modest degree of protection from all-cause and CVD mortality, but associations are more inconsistent for cancer mortality (5, 20). Fewer studies have been conducted in Asia, Africa, and Latin America. In countries with wide ethnic and dietary diversity, DP studies are associated with unique challenges.

Similar to *a priori* approaches, both FA and CA methodologies require many choices by the researcher: if the original dietary data should be collapsed further; if input variables should be standardized (Z-transformed) or energy adjusted; how many patterns to retain, report, and analyze; how to interpret and label patterns (see Table 3). The strengths and limitations of each DP approach and the subjective decisions made at many levels may all have consequences for subsequent analyses and the overall study interpretation. If published studies would improve the reporting of all decisions made during data handling and statistical analysis, the interpretation of *a posteriori* studies might be more efficient. Also, to clarify the utility of DPs in nutritional epidemiology, additional methodological research is needed, including validation and reproducibility studies (5, 20).

The many consistent DP findings across studies and methodologies, however, are important to health promotion. Rather than advocating avoidance or promoting individual nutrients, these findings support the role of a healthy overall dietary pattern in chronic disease prevention (5).

## Validity of DPs

Several researchers point out that the DP differences seen across populations and studies could be related to differences in dietary assessment (5, 11, 20, 34). Such differences may depend on the specific food habits in the examined populations, but could also depend on the relative validity of the dietary assessment methodology used. Since DP studies require information about the usual diet, single 24-h recalls (give episodic dietary information from specific days) or brief FFQs (intended for ranking individuals on intakes of selected food items) are not suitable dietary assessment methodologies (66). Also, it should be recognized that measurement errors, which may arise from the individuals’ ability to provide the requested dietary information, or from the format of the assessment tool (66, 67), will influence the emerging patterns and may contribute to inconsistences across studies.

However, several reviews aimed at evaluating the nutritional adequacy of DPs using objective biomarkers of dietary intakes concluded that, irrespective of approaches, the observed associations between DPs and biomarkers are generally in the expected directions (11, 20, 32, 34). This is especially so for micronutrients and nutrients found in vegetables and fruits (e.g. vitamin C, carotenoids, folate), but the micronutrients less likely to be adequately assessed are vitamin E (tocopherols) and B12 (32). Similarly, Kant reports that DPs consistent with notions of a healthy diet were associated with blood levels of vitamin C, folate, most carotenoids, as well as vitamin E in the expected directions (20). However, others report that the associations between DPs and plasma lipid profiles generally show a greater variability (11, 20).

Some reviewers suggest that if a dietary index does not predict mortality or morbidity of chronic disease, the index itself or the study design may need improvement (18, 33). Others suggest that dietary quality indices do predict mortality and morbidity, and thus could aid in further development and refinement of the dietary guidelines to promote health and well-being in the population (31). For instance, the HEI was developed as a single quality measure of diet quality; the original was a 10-component index based on the US Dietary Guidelines and the Food Guide Pyramid (30). Studies in two large US cohorts had indicated that the original HEI was inversely associated with CVD risk in men, but only a weak association was seen in women. The adjusted version AHEI, however, was inversely associated with moderate CVD risk in both men and women, but not with cancer risk. However, since the MDS has been associated with prediction of both CVD and cancer risk in the Greek population, it should be possible to identify index components that are more or less informative, and thus potentially help refine the guidelines (31).

## DPs and chronic disease

### Systematic reviews

Two papers were SRs of original DP research with focus on specific disease endpoints (60, 61), and one SR examined studies of the MedD and several health outcomes (62).

Brennan et al. conducted a systematic review and meta-analysis of DPs (defined by FA or PCA) and the risk of breast cancer (61). Among 73 potential articles, three reviewers using very specific criteria selected 16 articles (published 2001–2009) that identified prudent/healthy, Western/unhealthy or/and drinker DPs. The authors conducted pooled and sensitivity analyses, and tested for publication bias and heterogeneity among studies. In pooled analysis and in case-control studies, the Western DP was associated with increased breast cancer risk, but no significant associations were seen in cohort studies. In pooled analysis, a significant decreased risk was seen with the prudent DP. There was more heterogeneity among case-control studies with non-significant risk estimates, but less heterogeneity among cohort studies with significant protective associations with the prudent DP. The drinker DP was significantly associated with increased risk, and no evidence of heterogeneity was seen. One cohort study using a diet-history methodology (as opposed to FFQ) showed significant increased risk with the Western DP and decreased risk with the prudent DP (61).

Mente et al. systematically reviewed studies of the associations between dietary exposures and coronary heart disease (CHD) and myocardial infarction (MI) in 361 cohort studies and 51 RCT (60). This substantial review, using two independent reviewers and pooled analysis (including stratified analysis and tests of heterogeneity among studies), evaluated studies according to four of the Bradford Hill ‘criteria’ for causality: strength, consistency, temporality, and coherence. The authors concluded that there was strong evidence for a causal link (i.e. support from all four criteria) with CHD for several protective factors, including higher intake of vegetables and nuts; and Mediterranean and high-quality DPs. The characteristics of ‘high-quality diets’ were, however, not reported in detail. The evidence was also strong for harmful effects of intakes of trans-fatty acids and foods with high glycaemic index (GI) or glycaemic load (GL). In studies of high methodological quality, there was strong evidence for protection of mono-unsaturated fatty acids (MUFA) and of prudent DPs, and for adverse effects of Western DPs. Beneficial effects of the MedD were only observed in randomized clinical trials (RCT) (60).

The aim of the review by Sofi et al. (62) was to update a previous systematic review and meta-analysis (68) that investigated the effects of adherence to the MedD on several health outcomes. Two independent reviewers identified a total of 18 prospective cohorts using index scores to examine the MedD. The meta-analysis confirmed previous observations of convincing protective effects of the MedD on all-cause mortality, mortality from and incidence of CVD, the risk of neoplastic diseases, and the occurrence of neurodegenerative diseases; no significant heterogeneity was observed across studies.

In summary, the SRs using independent reviewers and strict criteria for inclusion and quality assessment indicate that the prudent DP, high-quality diets and the MedD (i.e. high consumption of vegetables, fruit, nuts, legumes, fish, vegetable oil, and low-fat dairy products) were associated with decreased risk of chronic diseases like breast cancer, CHD and MI, and of all-cause mortality. In contrast, DPs including high-fat and processed meats, refined grains, and sugar-rich products were linked to increased risk.

### Other reviews – general DPs

Kastorini et al. reviewed articles from cohort, cross-sectional and case-control studies, and clinical trials on DPs, the MedD, exposure to GL/GI and sub-types of fatty acids, and their associations with risk of type 2 diabetes mellitus (42) and concluded that dietary habits and obesity were the main risk factors for type 2 diabetes and that healthy DPs (like the MedD) were beneficial (42).

Lopez-Garcia et al. reviewed original research articles of trans-fatty acids, high GI/GL exposure, and DPs on the endothelial function (36). A previous review summarized dietary effects of fish oils, antioxidant vitamins, folic acid, and arginine (69). The combined conclusion was that endothelial dysfunction is an early marker of CVD risk, and that prudent DPs high in omega-3-fatty acids have beneficial influences on inflammatory and endothelial activation markers, but trans-fatty acids, high GL, and Western DPs have detrimental effects on endothelial function (36).

Srinath Reddy et al. reviewed the evidence of the link between diet and hypertension and CVD, and emphasized the need for critical appraisal of methodological issues when interpreting results and for public policies to facilitate lifestyle changes (37).

Giugliano et al. hypothesized that a dietary strategy that lowers CHD risk is also associated with lower generation of a pro-inflammatory milieu. After review of observational and intervention studies, the authors discuss that because DPs poor in natural antioxidants and fiber from plant foods may cause activation from the innate immune system, the lipid lowering effect (and reduced CHD risk) of low-fat diets may only be observed in prudent low-fat diets (3).

Schultze et al. reviewed 17 DP studies examining the risk of CHD and stroke using both *a priori* and *a posteriori* DP approaches and discussed benefits of the RRR. This review concluded that plant-based DPs rich in vegetables, fruits, and whole grains, but low in meat and refined grains and healthy sources of fat (e.g. vegetable oils, fish, and nuts) would be helpful in preventing CHD and stroke (39).

Baxter et al. reviewed DPs and metabolic or insulin resistance syndrome in three robust studies with large samples (38): The Isle of Ely, Malmö Diet and Cancer study, and the Cardia study. DPs rich in fruit and vegetables, dairy foods, and whole grain cereals showed protective associations while high meat intake had a negative health impact (38).

Edefonti et al. reviewed 19 articles (published 1995–2008) examining DPs and breast cancer incidence (41). The authors concluded that a diet rich in high-fat and high-sugar foods was generally associated with increased breast cancer risk, while DPs high in vegetables, fruits, fish, and poultry were associated with reduced breast cancer risk (41).

Randi et al. reviewed 32 articles (published 1992–2009) on DPs and colon-cancer risk (45) with a range of different study designs. High consumption of red and processed meats, refined grains, sweets, and alcohol and low consumption of fruits and vegetables, whole grain, fish, and poultry tended to be associated with increased colorectal cancer risk (45).

Miller et al. reviewed prospective cohort and population based case-control studies on DPs and colorectal cancer risk (44) and identified 16 articles (published 1992–2009) using different DP approaches. In men, higher scores of the dietary indices were consistently associated with decreased colorectal cancer risk, but associations were less conclusive in women. Fruits and vegetables were important components of all indices. Although the associations were modest and not consistently statistically significant across studies, the authors concluded that directions were in agreement with previous studies regarding colorectal cancer risk (44).

Since cross-sectional studies describe the prevalence of exposures and disease at the same point in time, the findings from three studies can only be suggestive. A review of six DP studies found that Western DPs tended to be associated with increased diabetes risk, and prudent DPs with reduced risk (40). A review of 30 DP studies among adults showed tendencies for high index scores to be inversely associated with high BMI or obesity (35). Similar conclusions were reached by a review of 11 cross-sectional DP studies among older adults (28).

In summary, although not entirely consistent across studies, these non-systematic review papers of DPs and chronic disease (i.e. regarding CVD, HT, CHD, metabolic syndrome, type 2 diabetes, and breast and colorectal cancer) reached similar conclusions as the SRs.

### Other reviews – MedD

Serra-Majem et al. identified 55 trials of MedD, but only 43 were reviewed (49) because of limited numbers of participants and insufficient dietary intervention designs. The important well-designed trials were the Lyon Diet Heart (70), the Indo Mediterranean Heart Study (71), the GISSI Prevention Trial for Secondary Prevention (72), and the Spanish study by Esposito et al. (73). These have shown clear reductions in CHD event rates, CVD mortality, or in CVD risk markers. A need for primary prevention trials was identified and emphasized (49).

Buckland et al. (2008) reviewed 21 studies of MedD and overweight and obesity (seven cross-sectional, three prospective cohorts and 11 intervention studies). About half of the eight studies conducted in non-Mediterranean countries suggested less obesity or weight loss with MedD. No prospective study showed increased obesity risk with MedD (51).

Kastorini et al. reviewed three prospective cohort studies, 11 cross-sectional studies, and 21 RCTs to evaluate if the protective effect of the MedD on CHD potentially is due to obesity or weight loss (56). In individuals following the MedD with energy restriction and/or physical activity, both bodyweight and CVD risk factors decreased, but comparisons were difficult across studies due to differences in design and country of origin. It was especially difficult to separate the effect of energy restriction and increased physical activity, from that of the dietary composition (56).

Verbene et al. reviewed observational studies using indexes to examine MedD and cancer (58). Seven cohort studies and five case-control studies (out of 62) met the inclusion criteria. The combined evidence suggests that the MedD protects against cancer in general, bur call for more research of selected cancer sites (58).

Also, Tyrovolas and Panagiotakos gave an overview of 10 studies (articles published 1985–2009) from seven countries examining associations between MedD and CVD, CHD, and cancer (57).

Kok and Kromhout focused on the diet consumed in Crete during the 1950s and early 1960s and discussed studies on CHD and mortality (46). In addition, Kokkinos et al. discussed and summarized the current understanding of how dietary factors (i.e. the dietary composition of the DASH diet or MedD) influence blood pressure (47). Bach et al. reviewed studies of several different indices used to evaluate adherence to the MedD from several countries (i.e. Greece, Denmark, China, Spain, other European countries, and Israel); healthy prudent pattern for health promotion coincides with that of a MedD pattern (48).

Esposito et al. reviewed the risk factors of the metabolic syndrome, and dietary intervention studies examining their effect on the metabolic syndrome (50) and concluded that a healthy dietary pattern like the MedD would reduce the risk of CVD and the metabolic syndrome. A brief review discussed the mechanisms by which the MedD would lower the risk of chronic inflammation and thereby metabolic diseases (52). Another review concluded that several components of the MedD have been inversely related to obesity, blood pressure, dyslipidaemia, and chronic inflammation (53).

A series of integrated case-control studies in Italy during the early 1990s examining MedD and cancer risk was reviewed in two papers (54, 55). Several characteristics of the MedD, like olive oil, whole grains, fish, and moderate consumption of red meat, affected cancer risk favorably.

In addition, Féart et al. updated the current knowledge concerning the MedD and cognitive function based on two prospective cohorts, where adherence to MedD was associated with reduced risk for mild cognitive decline (MCD), for conversion of MCD into Alzheimer's disease (AD), and for AD (59).

In summary, these non-systematic reviews of experimental and prospective studies indicate health benefits from MedD. Review articles with clear inclusion criteria that did not mix different study designs produced results more easy to interpret. Although not totally consistent, the majority of the non-systematic review articles were in agreement with the SR of MedD (62).

## Whole diet trials

Several articles described a couple of large trials that have received a lot of attention. An important design aspect of these two trials was that foods were provided to participants (5). The Lyon Heart Study, conducted in the late 1980s, was a randomized single-blinded secondary prevention trial aimed at preventing death and recurrent MI by dietary modification in survivors of a first MI (8). Participants, men and women below 70 years of age, were carefully instructed to adapt a MedD including more bread, more root and green vegetables, more fish, less meat (i.e. replace red meat like beef, lamb, and pork with poultry), and no day without fruit. Also, butter and margarine were replaced by a specific margarine, based on rapeseed oil, supplied by the study. Compared to olive oil, content of SFA and MUFA was similar (SFA 15% and MUFA 48% of total fatty acids), while the content of linoleic acid and alpha-linolenic acid was higher (2-fold; 16.4%, and 8-fold; 4.8%, respectively). After 27 months of follow-up, the rate of cardiac death and non-fatal infarction was clearly and significantly reduced in the experimental group, and after 4 years of follow-up, a 50–70% reduction in the risk of reoccurrence was documented in the experimental compared to the control group. Hence, the data confirmed the protective effect of the MedD (8).

Similarly, the DASH feeding studies were a series of controlled trials, among men and women aged 22 years or older (mean age 44 years), designed to test if whole diet modification had an effect on blood pressure (7, 13, 15, 74). The DASH diet emphasizes fruits, vegetables, and low-fat dairy products; includes whole grains, poultry, fish and nuts; and is reduced in dietary fats (e.g. saturated fat, SFA), red meat, sweets, and sugar-containing beverages (75). The most effective DASH diet was also reduced in salt (75). This salt reduced DASH diet (i.e. rich in potassium, magnesium and calcium and reduced in SFA and sodium) resulted in the lowest systolic blood pressure (reduction of systolic BP when compared to control group: 7.1 mmHg in normotensives and 11.5 mmHg in hypertensives). Since the effect of each dietary factor is typically modest, the best interpretation of this impressive reduction is that multiple dietary factors influence BP and that the influence of a combination of factors can be substantial (7). A review of the DASH-studies and two trials that used the DASH eating patterns [i.e. the small Diet, Exercise, and Weight Loss Intervention Trial (DEW-IT) and the multicenter PREMIER trial] concluded that a diet rich in fruits, vegetable, and low-fat dairy foods quickly and significantly reduces blood pressure, that the low sodium alternative had even greater effects, and that this diet also improves other CVD risk factors like total and LDL cholesterol (76). The PREMIER trial that was a community lifestyle intervention project for blood pressure control (i.e. not a feeding trial), had smaller effects than had the original DASH trials (77). In addition, the Cardiovascular Risk Reduction Dietary Intervention Trial (CRRDIT) was a 4-year series of multicenter randomized clinical studies to evaluate dietary intervention on men and women, aged 25 to 70 years, with elevated CVD risk factors (78). During the 10-week interventions meals that fulfilled the dietary recommendations were prepared and distributed to treatment participants. The CRRDIT demonstrated multiple and concurrent clinical benefits to persons at increased CVD risk, due to hypertension and dyslipidaemia and type-2 diabetes alone or in combination. A review of three trials (i.e. the Lyon Heart Study, DASH and CRRDIT trials), concluded that well-balanced diets that meet the recommended intakes of minerals, vitamins, and macro-and micronutrients recommended by the US Food and Nutrition Board of the National Research Council, and the nutrient intake recommendations from US national health organizations (79–81) will improve risk factors of CVD (64).

## Summary and conclusions

Although some inconsistencies were seen across the individual studies (potentially due to weaknesses in study design or to the decisions made by researchers when generating the DPs using both *a priori* and *a posteriori* methods), the systematic reviews regarding DPs and disease reached similar conclusions regardless of DP approach. Diets rich in plants foods like vegetables, fruits, nuts, and whole grain cereals, and with frequent use of fish, low-fat dairy products and vegetable oils, but low in refined cereal and sugar-rich products, and low in red and processed meats are associated with lower risk of a majority of the examined chronic diseases. Such DPs will also provide high amounts of micronutrients (essential minerals, vitamins, and fatty acids) and be rich in other bioactive components (e.g. antioxidants, phenolic compounds, phytoestrogens) associated with protection of several chronic diseases. Depending on the country of origin and study design, such diets have received different labels, e.g. DASH diet, Mediterranean diet, prudent pattern, High-Quality Diet (60–62), and Nordic Diet (82–85). The specific food choices may vary, but the overall dietary composition is similar.

The contrast to these plant food based diets is the Western pattern, which is common in westernized counties but spreading worldwide at a fast pace (86). Most of the studies using *a posteriori* approaches identified Western DPs as being related to increased risk of cardiovascular and metabolic diseases and to colon cancer. The specific food choices of the Western DP may vary slightly across populations, but the general characteristics are the dominance of red and processed meats, ‘junk foods’, refined cereals, confectionaries, sweets, sugar-containing beverages, and ultra-processed foods manufactured by the food industry. Biomarker studies have demonstrated that the Western DP is associated with low concentrations of micronutrients and likely provides less of other bioactive substances with health benefits found in plant foods. However, the Westernized DP also includes many processed and ultra-processed foods that likely contribute components with potential adverse health effects; those added during the manufacturing process (like trans-fatty acids) or formed during prolonged heat treatment (like heterocyclic amines, acryl amide, or advanced glycation/lipidoxidation end-products, AGE/ALE). Studies within EPIC (European Prospective Investigation into Cancer and Nutrition) indicate that the use of manufactured food products currently (i.e. data collected in the 1990s) is higher in northern European countries than in Mediterranean countries (87, 88). However, researchers argue that the traditional diets of the Nordic countries have important health advantages (89), and many projects currently examine the health influences of components of Nordic diets (89–91). Since the health impact of a modern healthy Nordic DP has only been examined to a limited extent so far (82–85), the EPIC observations emphasize the need to strengthen this line of research.

It should be noted that the identified review articles overlap. Since they review the same subject area during the same time period, it was expected that the same original research articles would be cited by many reviews. The conclusions should therefore not be interpreted in a cumulative sense. Several review papers identified that inconsistent results across studies were likely due to design weaknesses of the original research studies. Incomplete information also contributed to difficulties in interpreting study findings. It should be noted that in those articles where fuller study descriptions were provided and where design issues were not major obstacles, the overall conclusions were similar regardless of DP approach and specific disease end point. Only a few of the identified review articles were, in a strict sense, SRs (60–62), following processes similar to those defined by the guidelines formulated for the current NNR revision project (63), or the guidelines issued by the working group of Grading of Recommendations Assessment, Development and Evaluation (GRADE) intended for evaluating health research (www.gradeworkinggroup.org/), or those used in the report from the World Cancer Research Fund/American Institute of Cancer Research (WCRF/AICR), evaluating the link between food habits, nutrition, physical activity, and cancer risk (92). In such reviews, two or three independent reviewers follow strict inclusion, exclusion, and study quality criteria and comprehensively describe the process of selecting, grading, and judging their findings. In order to strengthen the knowledge base regarding food and DPs and chronic disease, not only is original research of high quality required, but also systematic reviews that follow structured processes so that studies are objectively selected and judged.
